# Coping with hypertensive treatment at Bono Regional Hospital in Sunyani, Ghana: a prospective observational cohort study

**DOI:** 10.11604/pamj.2023.45.185.39994

**Published:** 2023-08-25

**Authors:** Prince Owusu Adoma, Edward Wilson Ansah, Daniel Apaak, Richard Osei Agjei, Emmanuel Kumah, Richard Boateng, Elvis Enowbeyang Tarkang

**Affiliations:** 1 Department of Health Administration and Education, Faculty of Science Education, University of Education, Winneba, Ghana,; 2 Department of Health, Physical Education and Recreation, Faculty of Science Education, University of Cape Coast, Cape Coast, Ghana,; 3 Department of Population and Behavioural Science, University of Health and Allied Sciences, Ho, Ghana

**Keywords:** Coping strategy, blood pressure control, hypertension treatment, Bono Regional Hospital

## Abstract

**Introduction:**

the stress associated with hypertension treatment makes using coping strategies inevitable. However, most patients with hypertension apply inefficient coping strategies, leading to uncontrolled blood pressure (BP). The study analyzed coping strategies associated with hypertension treatment and determined how these coping strategies predicted the current BP of patients with hypertension.

**Methods:**

the study was a prospective observational cohort conducted between January and December, 2020. Consecutive sampling technique was used to enumerate 508 patients who consistently sought treatment at the healthcare facilities. A sphygmomanometer was used to measure BP to determine controlled and uncontrolled BP based on Ghana Health Service standards. A questionnaire was adapted from Coping Inventory for Stressful Situations-2 to measure patients' coping strategies. Descriptive statistics, cut off percentage and multiple linear regression were applied in analyzing the data at a 0.05 level of significance.

**Results:**

females were two-thirds (74%) of the study population and the mean age was 58.40 ± 11.72. All patients with hypertension used the three coping strategies: emotion-oriented coping (EOC), task-oriented coping (TOC) and avoidance coping (AC). However, EOC was highly used (61.2%), followed by TOC (58.5%) and AC (46.2%). Also, the study found coping with treatment regimens to be relatively poor since it was only physical exercise (79.5%) that they effectively observed. The multiple linear regression results revealed that the three coping strategies were significant predictors of current BP levels [F (3, 117) = 12.390 at p < 0.001]. Thus, AC, TOC, and EOC explained 37.4% of the variability of current BP status (R2 adj=0.374). Specifically, patients who use TOC (66.3%) were more likely to have a controlled BP than those using EOC (53.7%) and AC (35.8%).

**Conclusion:**

patients' coping strategies were inadequate for hypertension treatment since treatment regimens were poorly observed. Meanwhile, EOC is most likely to negatively affect a patient's treatment, leading to uncontrolled BP. Our study recommends the need to encourage patients to combine their EOC with TOC to enable them control their BP better.

## Introduction

The stressful nature of hypertension treatment makes usage of coping strategies inevitable, leading to the application of diverse coping strategies, of which some are inefficient, usually resulting in uncontrolled blood pressure (BP). Hypertension treatment is basically chronic in nature and requires lifestyle modifications for an effective outcome. Exposure to long-lasting stressful event has been hypothesized as a risk factor for high BP [1] and the effect of coping strategy on BP is still unclear [2]. Patients use two main coping strategies when subjected to varying degrees of stress, i.e., problem-focused strategy and emotion-focused strategy [3]. Problem-focused coping often has positive outcomes, whereas emotion-focused coping ends up being undesirable [4]. Generally, a problem-focused coping enables patients to implement problem-solving activities; and if the task is considered beyond their capabilities, they use their emotions. However, emotion-focused coping centers on emotions rather than the problem. Although most forms of emotional coping may lead to negative outcomes, evidence shows that emotional coping, such as religious coping, may have both positive and negative impacts on treatment [5,6].

Theoretically, Lazarus and Folkman's [7] coping theory demonstrates the complexity and numerous styles of managing stress. The coping theory describes the cognitive, and behavioural efforts used by individuals to deal with stressful events. In hypertension treatment, some stressors coincide with treatment regimens. For instance, a diet with a high intake of vegetables, fruits, and whole grains are recommended for hypertension treatment, but, patients are nonadherent [8] due to stress associated with a change in diet. A patient uses either cognitive or behavioural strategies to deal with such a situation. However, individuals are prone to use maladaptive strategies in managing the stress associated with their treatment. The Bono Region Hospital (BRH) annual report has showed a decline in patients with hypertensive seeking care over the years, i.e. from 1,014 in 2012 to 353 in 2016 [9]. However, there seems to be no published research to ascertain the reason associated with the persistent decline. This evidence corroborates Tenkorang and Kuuire's [10] study which indicated that hypertension treatment from sub-Saharan Africa is mixed, with those from Ghana scanty and limited. Therefore, we analysed coping strategies associated with hypertension treatment and determined how these coping strategies predicted the current BP of patients. Our research filled the gap in literature and the findings could serve as a guide to inform stakeholders and policymakers on effective coping strategies for hypertension treatment.

## Methods

### Study setting

Our research was carried out at a secondary level service provider, BRH in Sunyani, which is the capital city of the Bono Region. The Bono Region is situated in the middle belt of Ghana where the population is predominantly farmers. The hospital is a 400 ultramodern bed facility with the state of art equipment. It is also a government owned facility under Ghana Health Service (GHS) with the vision to meet community needs for healthcare for both the sick and healthy. It is the biggest facility in the region and serves as a referral centre. It has a special clinic (diabetic and hypertensive) for treating hypertension and treating the largest hypertensive population in the region.

### Study design and population

A prospective observational cohort design was used to carry out the study between January and December, 2020. The population were patients with hypertensive, defined as patients with a systolic pressure ≥ 141 mmHg and a diastolic pressure ≥ 91 mmHg. The population included participants who were 18+ years; diagnosed of hypertension by a professional practitioner; and have continuously sought treatment for the past six months at BRH, prior to data collection. However, the study excluded pregnancy-induced hypertension, contraceptive pills/injectables or hormone replacement-induced hypertension and patients with psychiatric illness, and most importantly, those who refused to sign/thump print the consent form.

### Sampling technique and size

The hospital had 657 registered patients with hypertensive, however, 96 of the patients could not be traced, whereas 41 were not consistent with their treatment. We considered all the remaining 520, but, 508 (return rate of 98%) patients finally participate in the study. Since it was a complete enumeration, the study used consecutive sampling method to select all the patients.

### Instrument

A questionnaire developed from a pre-existing Coping Inventory for Stressful Situations-21 (CISS-21) Questionnaire [11] was used for the study. Items from CISS-21 were adapted to measure coping strategies in three categories: TOC, EOC, and AC. The draft questionnaire contained 38 items in four sections (A, B, C and D). Section A was on socio-demographic characteristics of patients, B measured coping strategies in the three categories, section C was on treatment regimen and section D reported on the current BP score of the patients. The draft instrument was reviewed to ensure validity and reliability. The instrument produced Cronbach's Alpha reliability coefficient scores of 0.80, 0.91, and 0.88 for TOC, AC, and EOC, respectively.

### Data collection procedure

We recruited our participants from the Diabetic and Hypertensive Clinic (Clinic 5) at the BRH, where hypertension cases are usually treated. We hired three research assistants (RAs) who helped in data collection. The RAs were BSc. Nursing graduates who were on their compulsory national service duties at the hospital but not at Clinic 5. They had no relationship with the patients; they were judged to be objective; and have no influence on the patients. The RAs were trained on administering the instrument and translating items into Twi Language, the local language among the Akan tribe in Ghana, for those patients who could not read or write English Language. Data collection was done at Clinic 5, and all patients signed/thump printed the consent form before taking part. The Institutional Review Board of University of Cape Coast approved the study. The BP of patients recruited for the study were first measured using the sphygmomsnometer and recorded by the research assistants. The BP readings of the patients were taken three times and the best reading was recorded. The readings were taken while the patients were seated comfortably with their arms placed closed to their chest. In addition, we distributed the questionnaire to all qualified patients, and they responded to a Likert scale on five-point scale, ranging from strongly disagree to strongly agree (1 to 5), with high scores signaling higher use of a coping strategy. Data collection took 12 months; the first six months (from January to June, 2020) were used to identify patients who consistently sought treatment at BRH, whereas the next six months (July to December, 2020) were used for data collection. The major problems encountered at the field were time constraints, and unfriendly nature of some of the patients. Also, only one patient felt too tired and weak to complete the questionnaire, and that questionnaire was excluded resulting in 508 data points.

### Method of data analysis

Statistical software, SPSS version 24.0 was used for data processing. First, we screened the data to remove duplicates, and fixed structural errors. The screened results indicated that there were no missing values, data was normally distributed and heterogeneous in nature. The mean systolic pressure of 120 - 140 mmHg and diastolic pressure of 80 - 90 mmHg was set as controlled BP, whereas uncontrolled BP was a systolic pressure ≥ 141 mmHg and a diastolic pressure ≥ 91 mmHg. A cut off percentage of 80% was set to measure effectiveness of coping with treatment regimens [12,13]. A multiple linear regression was calculated to predict the effect of coping strategies on current BP of the patient at statistical significant level of p < 0.05. The study reported F-statistic, a measure of the overall significance of the regression model, degrees of freedom and adjusted coefficient of determination (R2), a measure of how well the regression model fits the data.

## Results

### Socio-demographic characteristics of patients

For this study, 508 hypertensive patients from BRH were recruited. Table 1 indicated that females (74%) were three-fold of males (26%). The mean age was 58.40 (SD = ± 11.72) and most of the patients were in their late adulthood. Also, majority had attained a basic education (52.8%). A good number of the patients were married (68.1%) and employed (65.0%). Almost all the participants were Christians (90.4%), and about 41.9% diagnosed of hypertension when they were between 41 - 50 years. Most of the patients had other chronic-noncommunicable diseases: 69.4% had diabetes, 57.6% had stroke, while 48.5% had both stroke and diabetes. However, 28.5% of the patients reported no co-morbidities. Large portion of the patients had uncontrolled BP and the mean systolic pressure stood at 149 mmHg and diastolic was at 97 mmHg.

**Table 1:** socio-demographic characteristics of hypertensive patients

Item		Frequency	Percent (%)
Gender	Male	132	26.0
	Female	376	74.0
Age groups	Mean age	58.40	
	Standard deviation	± 11.72	
	<30 years	24	4.7
	31-40 years	32	6.3
	41-50 years	159	31.3
	51-60 years	154	30.3
	+61 years	139	27.4
Educational level	No formal education	136	26.8
	Basic education	268	52.8
	Secondary education	82	16.1
	Tertiary education	22	4.3
Marital status	Unmarried	162	31.9
	Married	346	68.1
Employment status	Unemployment	107	21.1
	Employed	330	65.0
	Housewife	20	3.9
	Pensioned	51	10.0
Religion	Christianity	459	90.4
	Islam	47	9.3
	Traditional religion	2	0.4
Age diagnosed of hypertension	<30 years	40	7.9
	31-40 years	79	15.6
	41-50 years	213	41.9
	51-60 years	164	32.3
	+61 years	12	2.3
Forum first diagnosed of hypertension	BAR hospital	350	68.9
	Other hospital	149	29.3
	Screening/Educational program	8	1.6
	Can’t remember	1	0.2
Length of time sought for hypertension treatment	<6 months	187	36.8
	6 – 12 months	173	34.1
	1 – 2 years	63	12.4
	2 – 5 years	42	8.3
	5 – 10 years	27	5.3
	+ 10 years	10	2.0
	Can’t remember	3	0.6
Co-morbidities	Diabetes	252	69.4
	Stroke	209	57.6
	Renal dysfunction	124	34.2
	ISH	84	23.1
	CHD	47	12.9
	Diabetes & Stroke	176	48.5
	None	145	28.5
Hypertension status	Controlled	354	69.7
	Uncontrolled	154	30.3
Mean BP	Systolic	149 mmHg	
	Diastolic	97 mmHg	

* N = 508; ISH – Ischemic Heart Disease; CHD – Coronary Heart Disease

### Coping strategies used by hypertensive patients

The results showed that all the patients used the three coping strategies (AC, EOC, and TOC) as part of their treatment. The three coping strategies were assessed separately from each other. The results indicated that 61.2% of the patients used EOC, 58.5% used TOC, and 46.2% used AC (Table 2). Dimension of EOC showed that almost all the patients (98%) agreed to focus on God as their healer for their treatment. Also, most patients (78.3%) agreed that they took some corrective actions immediately they were diagnosed with hypertension.

**Table 2:** coping strategies used by hypertensive patients

	Agree	Disagree	Rank	Total
	Freq. (%)	Freq. (%)		Freq. (%)
Emotion-oriented Coping	311 (61.2)	197 (38.8)	1	508 (100.00)
Focus on my God as my healer	498 (98.0)	9 (1.8)	1	507 (99.8)
Feel anxious about not being able to cope	384 (75.6)	123 (24.4)	2	508 (100.0)
Wish that I could change what had happened or how I felt	283 (55.7)	225 (44.3)	3	508 (100.0)
Become very upset	279 (54.9)	229 (45.1)	4	508 (100.0)
Blame myself for having gotten into the situation	113 (22.2)	395 (77.8)	5	508 (100.0)
Task-oriented Coping	295 (58.5)	209 (41.5)	2	504 (99.3)
Take corrective action immediately	393 (78.3)	109 (21.7)	1	502 (98.8)
Focus on the problem and decided on how I can solve it	315 (62.0)	193 (38.3)	2	508 (100.0)
Think about my life>	299 (59.6)	203 (40.4)	3	502 (98.8)
Analyse my problem before reacting	257 (50.6)	251 (49.4)	4	508 (100.0)
Work to understand the situation	212 (42.2)	290 (57.8)	5	502 (98.8)
Avoidance Coping	234 (46.2)	273 (53.8)	3	507 (99.95)
Praying to God	429 (84.4)	79 (15.6)	1	508 (100.0)
Time with special persons	226 (44.6)	281 (55.4)	2	507 (99.8)
Eat my favorite food/snack	216 (42.5)	292 (57.5)	3	508 (100.0)
Phone a friend	162 (31.9)	346 (68.1)	4	508 (100.0)
Go for vacation	137 (27.0)	371 (73.0)	5	508 (100.0)

N = 508

### Coping with treatment regimens

**Figure 1:**
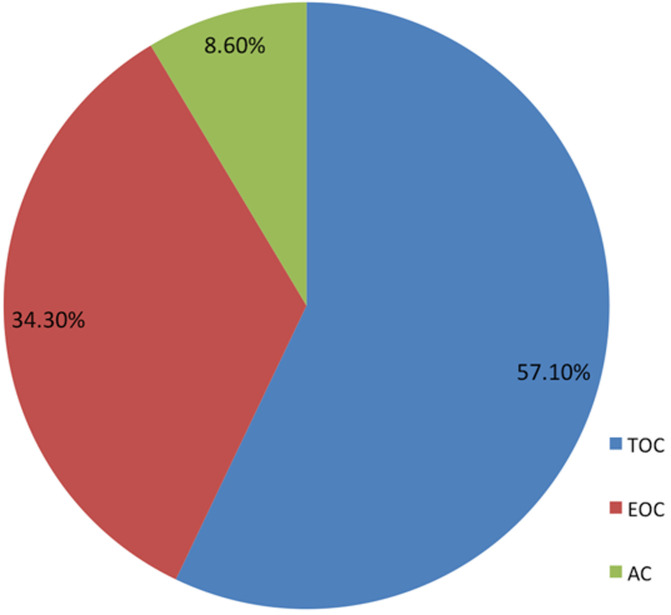
coping with engaging in physical exercise

**Figure 2:**
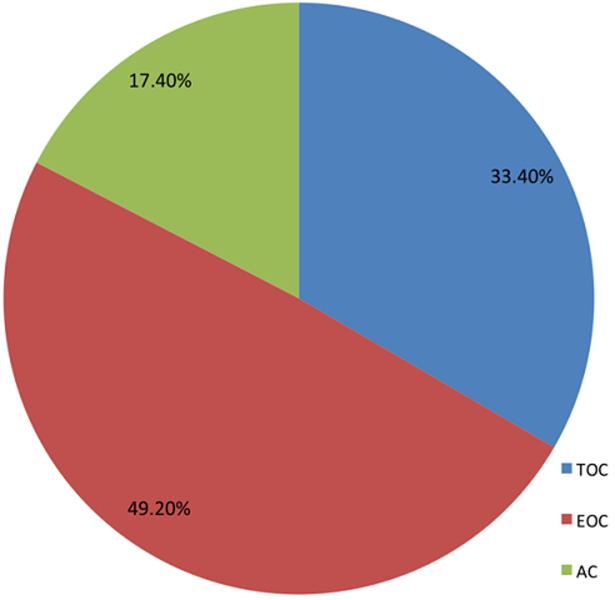
coping with taking diet low in salt

**Figure 3:**
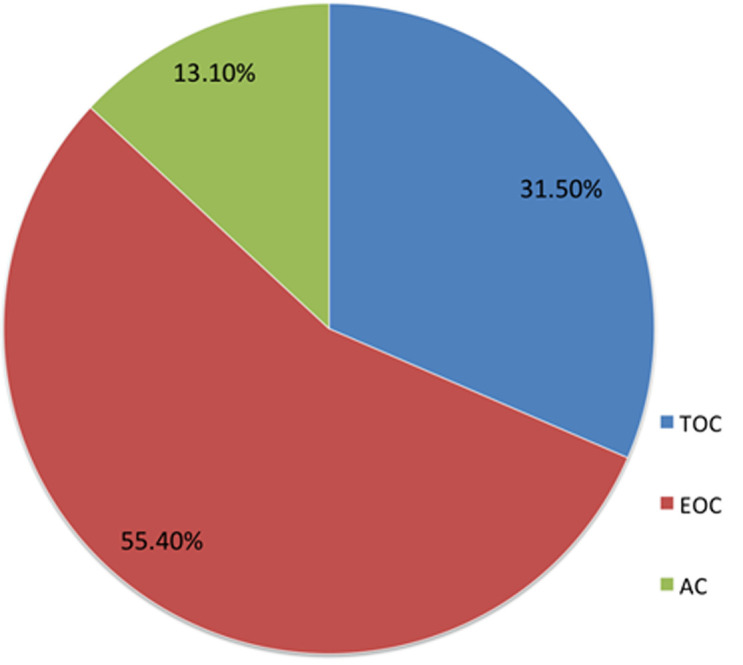
coping with meeting appointment

**Figure 4:**
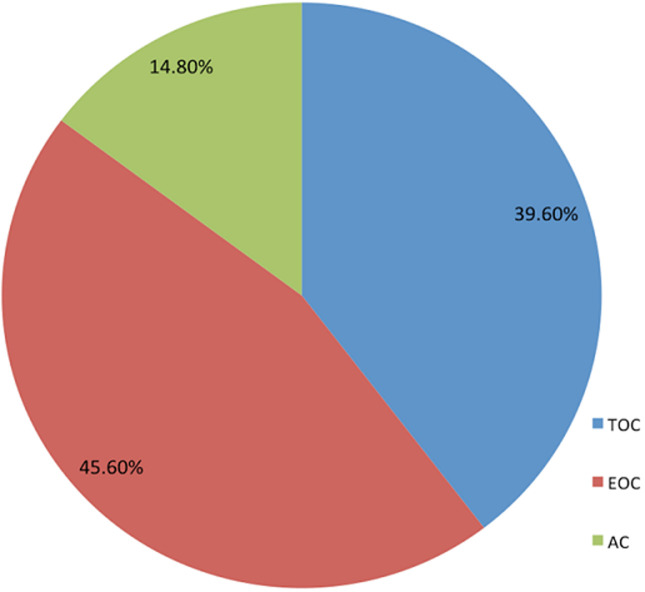
coping with antihypertensive medication intake

Patients treated their hypertension by undertaking physical exercise, consuming diet low in salt, meeting appointment and taking antihypertensive medications. Table 3 indicated that almost all the patients poorly coped with their treatment regimens, except for physical exercise. Statistically, patients effectively coped (79.5%) with physical exercise, at M = 1.20 and SD = 0.40. Meanwhile, two-thirds of the patients (66.5%) coped with diet low in salt. Unfortunately, only one-third (39.6%) effectively took their antihypertensive medication as prescribed. In relation to the specific coping strategy used for each treatment regimen, Figure 1 displayed that for physical exercise, patients made use more of TOC (57.1%) than all the other coping strategies. However, there was high preference for EOC (49.2%) for consuming diet low in salt as compared to TOC and AC (Figure 2). In relation to patients' appointment making and antihypertensive medications (Figure 3 and Figure 4), EOC was preferable, 55.4% and 45.6% of the patients used EOC, respectively.

**Table 3:** adherence to hypertension treatment

	Freq. (%)	Freq. (%)		
Physical exercise	404 (79.5)	104 (20.5)	1.20	0.40
Salt reduction	338 (66.5)	170 (33.5)	1.33	0.47
Meeting appointment	282 (55.5)	226 (44.5)	1.44	0.50
Medication intake	201 (39.6)	305 (60.4)	1.60	0.49

N = 508; Cut off percentage = 80.0%

### Influence of coping strategies on patients' current BP

The study findings showed that AC, TOC, and EOC significantly predicted the current BP levels of the patients (F (3, 117) = 12.390 and p < 0.001). Thus, AC, TOC, and EOC explained 37.4% of the variability of the current BP status of the patients (*R^2^ adj* = 0.374). The multiple regression analysis further revealed that AC, TOC and EOC individually predicted current BP levels of the patient at 0.05 significance level: t = 3.455, p = 0.001, SE=1.505; t = 4.927, p = 0.001, SE=2.248 and t = -2.326, p = 0.022, SE=1.756, respectively.

Furthermore, the beta coefficients value reveals the degree of effectiveness of variables based on the degree of influence; TOC (β=0.663), EOC (β=-0.537) and AC (β=0.358). In terms of multi-collinearity among the variables, inspection of the variance inflation factors (VIF) values of TOC, EOC and AC were critically considered if there were issues. According to Johnston *et al*. [14], a VIF value greater or equal to 2.5 indicates considerable collinearity. However, in our case, the VIF values range from 1.137 to 1.900, which is less than 2.5, indicating that there are no issues with multi-collinearity between the variables [14]. The standardized beta coefficient showed that TOC (66.3%) had the strongest influence on current BP levels followed by EOC (53.7%) and AC (35.8%). Table 3 illustrates that TOC has a positive and statistically significant influence on current BP level. Thus, if all other factors remain constant, a percentage change in TOC will result in 66.3% improvement in current BP level of the patients. However, EOC has a negative and statistically significant influence on current BP levels, thus, holding all other factors constant, a percentage change in EOC will result in a complete rise in current BP levels of the patients, raising their BP levels by about 53.7%.

**Table 4:** regression of coping strategies on controlled BP

Model	B	Std. Error	Beta	t	Sig.	Tolerance	VIF
Constant	82.546	4.036		20.451	< 0.001		
AC	5.201	1.505	0.358	3.455	< 0.001	0.880	1.137
TOC	11.073	2.248	0.663	4.927	< 0.001	0.581	1.722
EOC	-4.085	1.756	-0.537	-2.326	< 0.022	0.526	1.900

N = 508

## Discussion

Our study analysed coping strategies associated with hypertension treatment and determined how coping strategies predicted current BP levels of patients with hypertension. The results indicated that all patients used the three coping strategies (AC, TOC, and EOC) as part of their treatment. Using the three coping strategies means that patients applied different cognitive and behaviour efforts in managing their conditions, therefore, confirming the applicability of coping theory in hypertension treatment. The study finding is also similar to Ariff *et al*. study [15], which affirmed the use of significantly different coping styles to treat hypertension. Also, Ersek, Turner, and Kemp's study [16] described the use of different coping styles such as task persistence, avoidance coping, self-statement, asking for assistance, and relaxation as part of managing hypertension. The adoption of more than one coping strategy in hypertension treatment suggests that coping is not an event, but instead a process that shows how patients adjust to different situations. During the phase of the treatment, patients may change between the use of TOC, AC, and EOC strategies to enable them cope well with the stress associated with treatment. The use of different coping strategies has different consequences on treatment outcomes. For instance, ineffective coping may lead to non-adherence, uncontrolled BP, and probably dropout from treatment and associated complications. Moreover, the ability to use appropriate coping strategies is shaped by personal experiences and the outcome of the treatment [17].

In relation to patients' preferred coping strategies, unexpectedly, our study showed that patients preferred EOC to TOC and AC. Meanwhile, most studies reported the use of TOC among patients than EOC [2,4,9,10]. The inconsistency could be the result of the dynamics of the selected population, which comes with differences in personality and differences in styles, including coping strategies used. The preference for a particular coping strategy may be attributed to a belief that each person has their coping style, which may be relatively stable over time and across different stressful situations [18]. Thus, the ability to fit a particular coping strategy to specific and appropriate demands of different situations shows a high level of flexibility that is more likely to help than rigidity in coping. Most patients might have used EOC than TOC because it is focused on regulating stress associated with hypertension treatment [19]. This type of coping may be helpful if the stressor is something that cannot be changed. The application of EOC in hypertension treatment may have negative consequences on patients' treatment outcomes. Rahman *et al*. [20] observed that anxious patients found it challenging to regulate their BP. EOC is mainly considered a maladaptive coping strategy in hypertension treatment, which might have explained why BP targets are challenging to achieve in Ghana. For example, Sanuade *et al*. [21] found that out of 67.6% of participants who were aware of their hypertension status and on treatment, only 11.6% had their condition controlled. Also, it might have explained why there has been a high dropout rate among hypertensive patients at BRH.

Furthermore, our study found that nearly all the patients poorly coped with their treatment regimens, except for patients who engaged in physical exercise. Generally, literature on coping with specific treatment regimen is missing. However, the few existing studies found were consistent with our study finding. Patients might have been engaging in physical exercise since it formed part of their daily activity. Generally, people engage in household chores that require walking, squatting, stretching and other forms of physical exercise. Patients might find it as part of their normal daily activity, although, they might have not deliberately engaged in any form of physical exercise. Also, it could be that patients were educated about the importance of physical exercise on hypertension treatment. We speculate that poorly coping with diet low in salt, meeting appointment and taking antihypertensive medications is due to financial problems and lack of decision-making power. For instance, most patients were females who may have inadequate financial resources or probably lack decision making power on what to eat, and/or take antihypertensive medications. These decisions may be subject to their husbands'/caregivers' approval. Moreover, there is no functional system of reminder at BRH for appointment making for patients and for that matter it is likely that some may forget to honour their appointment.

Our findings also revealed that AC, TOC, and EOC significantly predicted the current BP levels of the patients with hypertension. However, TOC had the most substantial influence on the current BP levels of the patients as compared to AC and EOC strategies. Our study finding is congruent with other prospective studies that found that participants with high TOC scores showed significantly lower BP values [4,9,22]. A multiple logistic regression analysis on hypertension status showed that a high EOC and low TOC scores remain significant factors in determining BP state of patients [19]. Furthermore, coping strategies in the positive domain (problem-solving and social contact) were associated with lower BP, whereas those in the negative domain are connected with raised BP [4]. The findings imply that adaptive coping such as TOC, lowers BP values, whereas maladaptive coping such as EOC and AC, could elevate BP levels of patients. Also, studies revealed that participants with high TOC scores showed a significantly lower risk of hypertension than those with lower scores [2,4]. The studies attest that patients who used TOC strategies potentially adapt well and effectively reduce the stress associated with treatment, thereby achieving controlled BP.

The current study had some limitations. Nearly, all the hypertensive patients had comorbid conditions, and that could influence their BP status. Also, considering the sensitive nature of hypertension treatment, there is likelihood that there was under reporting, misrepresentation and misinformation during data collection since it was done at the hospital premises which could raise their current BP. Even though the study provided detailed analysis of coping strategies used by patients and how they influenced current BP levels, it may not be an entirely accurate measure for generalization since it was a facility-based.

## Conclusion

Our study assessed coping strategies used by hypertensive patients and determined how those coping strategies influenced patients’ current BP levels, at BRH, Sunyani. Hypertension treatment can be very stressful, leading to the application of varying coping strategies. Meanwhile, coping may have either positive or negative impact on treatment depending on the type of coping strategy used. Thus, inappropriate coping such as AC and EOC are likely to generate poor treatment outcomes. The application of appropriate coping strategies helps patients to manage the uncertainties and stress associated with their treatment. It is important that generalization of the study findings is done with caution since it was a facility-based study which is not necessarily representative of the hypertension patients community. Consequently, this study helps patients effectively adjust to the condition's demands and its treatment regimen. Therefore, there is a need to encourage patients to develop and devise ways of improving their coping skills. Also, patients should pay attention to the psychosocial aspect of their treatment to cope effectively with lifestyle changes associated with hypertension treatment. There is a need to motivate patients to learn how to use religious coping strategies positively and take proactive steps to promote such positive coping strategies for better treatment outcomes and quality of life.

### What is known about this topic

- *Literature posits that every chronic condition such as hypertension comes with psychosomatic pain that requires coping mechanism since patients adjust to different situations by applying different cognitive and behaviour efforts in managing their conditions;*
- *It is also known that there is a relationship between coping strategies and BP, however, it is still unclear the effect coping strategies have on patients BP;*
- *The use of appropriate coping strategies is shaped by personal experiences with hypertension, and the outcome of the treatment may be beneficial, whereas maladaptive coping may lead to non-adherence, poor treatment outcomes, and dropout from treatment*.

### What this study adds

- *Our study shows that all hypertensive patients used different coping strategies as part of treating their condition; unexpectedly, our study participants preferred emotional-oriented coping than task-oriented and avoidance coping strategies;*
- *The study also found that patients coping with treatment regimens was emotionally based and relatively poor since it was only physical exercise that they effectively observed compared to coping with antihypertensive medication, meeting appointment and taking diet low in salt;*
- *The multiple linear regression analysis revealed that avoidance coping, task-oriented coping and emotional-oriented coping significantly predicted the current BP levels of the patients where TOC has the most substantial influence on the current BP level of the patients*.

## References

[1] Rosendaal NTA, Hendriks ME, Verhagen MD, Bolarinwa OA, Sanya EO (2016). Costs and cost-effectiveness of hypertension screening and treatment in adults with hypertension in rural Nigeria in the context of a health insurance program.

[2] Sithu A, Ramli M, Jamalludi AR, Azarisman SM, Aszrin A (2018). Relationship between coping mechanisms to psychosocial stress with blood pressure in young adults: A pilot study. Bangladesh J Med Sci.

[3] Graven LJ, Grant JS (2013). Coping and health-related quality of life in individuals with heart failure: an integrative review. Heart Lung.

[4] Aina FO, Ajayi EA, Kumolalo FB, Inubile AJ (2016). Coping strategies and blood pressure control among hypertensive patients in a Nigerian tertiary health institution. J Dent Med Sci.

[5] Cozier YC, Yu J, Wise LA, VanderWeele TJ, Balboni TA, Argentieri MA (2018). Religious and Spiritual Coping and Risk of Incident Hypertension in the Black Women's Health Study. Ann Behav Med.

[6] Teteh DK, Lee JW, Montgomery SB, Wilson CM (2020). Working Together with God: Religious Coping, Perceived Discrimination, and Hypertension. J Relig Health.

[7] Lazarus RS, Folkman S (1984). Stress, appraisal and coping.

[8] Leme ACB, Hou S, Fisberg RM, Fisberg M, Haines J (2021). Adherence to Food-Based Dietary Guidelines: A Systemic Review of High-Income and Low-and Middle-Income Countries. Nutrients.

[9] Brong Ahafo Regional Hospital Annual Report for 2016.

[10] Tenkorang EY, Kuuire VZ (2016). Noncommunicable Diseases in Ghana: Does the Theory of Social Gradient in Health Hold?. Health Educ Behav.

[11] Calsbeek H, Rijken M, Bekkers MJTM, Henegouwen GPB, Dekker J (2006). Coping in adolescents and young adults with chronic digestive disorders: Impact on school and leisure activities. Psychol Health.

[12] Nguyen TM, La Caze A, Cottrell N (2014). What are validated self-report adherence scales really measuring?: a systematic review. Br J Clin Pharmacol.

[13] Sackett DL, Haynes RB, Gibson ES, Taylor DW, Roberts RS, Johnson AL (1978). Patient compliance with antihypertensive regimens. Patient Couns Health Educ.

[14] Johnston R, Jones K, Manley D (2018). Confounding and collinearity in regression analysis: a cautionary tale and an alternative procedure, illustrated by studies of British voting behaviour. Qual Quant.

[15] Ariff F, Suthahar A, Ramli M (2011). Coping styles and lifestyle factors among hypertensive and non-hypertensive subjects. Singapore Med J.

[16] Ersek M, Turner JA, Kemp CA (2006). Use of the chronic pain coping inventory to assess older adults' pain coping strategies. J Pain.

[17] Nyaaba GN, Agyemang C, Masana L, de-Graft Aikins A, Beune E, Larrea-Killinger C (2019). Illness representations and coping practices for self-managing hypertension among sub-Saharan Africans: A comparative study among Ghanaian migrants and non-migrant Ghanaians. Patient Educ Couns.

[18] Lazarus RS (1993). Coping theory and research: past, present, and future. Psychosom Med.

[19] Amnie AG (2018). Emerging themes in coping with lifetime stress and implication for stress management education. SAGE Open Med.

[20] Rahman AR, Wang JG, Kwong GM, Morales DD, Sritara P, Sukmawan R, all members of the Asian Cardiovascular Expert Forum Committee (2015). Perception of hypertension management by patients and doctors in Asia: potential to improve blood pressure control. Asia Pac Fam Med.

[21] Sanuade OA, Boatemaa S, Kushitor MK (2018). Hypertension prevalence, awareness, treatment and control in Ghanaian population: Evidence from the Ghana demographic and health survey. PLoS One.

[22] Mucci N, Giorgi G, De Pasquale Ceratti S, Fiz-Pérez J, Mucci F, Arcangeli G (2016). Anxiety, Stress-Related Factors, and Blood Pressure in Young Adults. Front Psychol.

